# High prevalence of methicillin resistance and PVL genes among*Staphylococcus aureus* isolates from the nares and skin lesions of pediatric patients with atopic dermatitis

**DOI:** 10.1590/1414-431X20154221

**Published:** 2015-05-08

**Authors:** F.S. Cavalcante, E.D. Abad, Y.C. Lyra, S.B. Saintive, M. Ribeiro, D.C. Ferreira, K.R.N. dos Santos

**Affiliations:** 1 Departamento de Microbiologia Médica, Instituto de Microbiologia Paulo de Góes, Universidade Federal do Rio de Janeiro, Rio de Janeiro, RJ, Brasil; 2 Instituto de Puericultura e Pediatria Martagão Gesteira, Universidade Federal do Rio de Janeiro, Rio de Janeiro, RJ, Brasil; 3 Centre for Ecological and Evolutionary Studies (Microbial Ecology), Faculty of Mathematics and Natural Science, University of Groningen, Groningen, The Netherlands; 4 Programa de Pós Graduação em Odontologia, Universidade Estácio de Sá, Rio de Janeiro, RJ, Brasil

**Keywords:** Atopic dermatitis, *Staphylococcus aureus*, Nasal colonization, Skin lesions, SCORAD

## Abstract

*Staphylococcus aureus* is highly prevalent among patients with atopic dermatitis (AD), and this pathogen may trigger and aggravate AD lesions. The aim of this study was to determine the prevalence of *S. aureus* in the nares of pediatric subjects and verify the phenotypic and molecular characteristics of the isolates in pediatric patients with AD. Isolates were tested for antimicrobial susceptibility, SCC*mec*typing, and Panton-Valentine Leukocidin (PVL) genes. Lineages were determined by pulsed-field gel electrophoresis and multilocus sequence typing (MLST). AD severity was assessed with the Scoring Atopic Dermatitis (SCORAD) index. Among 106 patients, 90 (85%) presented *S. aureus* isolates in their nares, and 8 also presented the pathogen in their skin infections. Two patients had two positive lesions, making a total of 10 *S. aureus*isolates from skin infections. Methicillin-resistant *S. aureus*(MRSA) was detected in 24 (26.6%) patients, and PVL genes were identified in 21 (23.3%), including 6 (75%) of the 8 patients with skin lesions but mainly in patients with severe and moderate SCORAD values (P=0.0095). All 24 MRSA isolates were susceptible to trimethoprim/sulfamethoxazole, while 8 isolates had a minimum inhibitory concentration (MIC) to mupirocin >1024 μg/mL. High lineage diversity was found among the isolates including USA1100/ST30, USA400/ST1, USA800/ST5, ST83, ST188, ST718, ST1635, and ST2791. There was a high prevalence of MRSA and PVL genes among the isolates recovered in this study. PVL genes were found mostly among patients with severe and moderate SCORAD values. These findings can help clinicians improve the therapies and strategies for the management of pediatric patients with AD.

## Introduction

Atopic dermatitis (AD) is a chronic inflammatory skin disease (1) that affects 10-20% of children worldwide (2). Several indexes have been proposed to assess AD severity; however, SCORAD (scoring atopic dermatitis) is the most widely used index (3). It adds a point for each symptom such as extension of eczema, dryness, pruritus, sleep disturbance, etc. Patients who score >25, 25-50, or >50 are considered to have mild, moderate, and severe AD, respectively.

Genetic predisposition, skin barrier defects, and environmental exposure are considered to be associated with the development of AD (4). Skin colonization by *S. aureus* may also contribute to the onset and/or aggravation of lesions (5) because staphylococcal toxins such as Panton-Valentine Leukocidin (PVL) and superantigens can aggravate the eczema (6). The prevalence of *S. aureus* in AD patients is up to 80% in nasal colonization (7-9) and can vary from 75% to 100% in skin lesions (8-11), while for methicillin-resistant *S. aureus* (MRSA), the prevalence ranges from 0.5% to 16% (7-10,12,13).

The resistance to methicillin in *S. aureus* is encoded in a*Staphylococcal* chromosome cassette *mec*(SCC*mec*), of which there are 11 types. Among AD patients, MRSA isolates usually carry *mec* cassettes commonly found in healthy individuals from the community, such as types IV and V (11). The genetic profile of most MRSA recovered from AD children belongs to well-established community lineages of different geographical regions such as ST188 in Korea (11).

Although some studies have reported *S. aureus* prevalence in AD patients, as well as the detection of the bacterial virulence factors and their relation with clonality (7,9,11), this has not yet been analyzed in Brazil. Therefore, this study aimed to verify the prevalence of *S. aureus* colonization, including MRSA, in pediatric outpatients with AD and characterize the SCC*mec* types, PVL genes, and clonality of isolates from nares and AD skin lesions. In addition, we correlated the presence of PVL genes with disease severity and isolate characteristics.

## Material and Methods

### Setting and study populations

A cross-sectional study was conducted between September 2011 and September 2012 at the Universidade Federal do Rio de Janeiro hospital pediatric dermatology outpatient clinic, which provides assistance to about 130 AD pediatric patients. The target population of the study included patients diagnosed with AD of both genders who were 16 years old or less. The population was predominantly low income. The study was approved by the Ethics Committee of Instituto de Pediatria e Puericultura Martagão Gesteira, Universidade Federal do Rio de Janeiro (#51/11).

### Collection and bacterial isolates

Swabs from the anterior nares and infected skin lesions were obtained from 106 patients. All infected skin sites of skin with infection were analyzed. Specimens were cultured on mannitol salt agar (Oxoid, UK) and characterized with standardized tests (14). The following controls were used: *S. aureus* strains ATCC 25923 and ATCC 29213 (for susceptibility tests), Mu50 (SCC*mec* type II) (15), and the clinical isolates described previously (SCC*mec* types II, III and IV; PVL genes positive) (16).

### Antimicrobial susceptibility tests

All isolates were submitted to diffusion testing (17) and minimum inhibitory concentrations (MICs) for oxacillin and vancomycin by microdilution broth (18). The MIC test for mupirocin was performed by the E-test^¯^(BioMérieux, France) in all mupirocin-resistant isolates.

### Characterization of SCCmec type and detection of PVL-encoding genes

Bacterial DNA was extracted (19), and all*S. aureus* isolates were submitted to polymerase chain reaction (PCR) for PVL genes (20). SCC*mec* typing was performed on all MRSA isolates (21).

### PFGE, RM test, and MLST

Eighteen *S. aureus* isolates from 8 patients who presented at least two clinical sites positive for the pathogen were evaluated for genotypes by pulsed-field electrophoresis (PFGE) (22). This technique is based on DNA fragmentation followed by electrophoresis. Each isolate submitted to PFGE shows a band pattern. The isolates were grouped in clones according to band patterns similarities (23), and the clonality was obtained by comparisons with previously published images (24). A restriction-modification (RM) test was carried out to determine the bacterial clonal complexes (25). Isolates that were not included in a clonal complex by the RM test were submitted to the multilocus sequence typing (MLST) method (26).

### Statistical analysis

All data were analyzed using the SPSS 20.0 software program for Windows (SPSS Inc., USA). The exact Fisher's test and chi-square test were used to compare data. Significance was established at 5% (P<0.05).

## Results

### Bacterial isolates

Nasal and skin lesion swabs were collected from 106 AD patients. Ninety (85%) patients presented *S. aureus* isolates in nares, and 8 (7.5%) also presented the pathogen in their skin infections. Two patients had two infected lesions positive for *S. aureus*, for a total of 10*S. aureus* isolates from skin infections. Among 90 patients with *S. aureus* isolates, 24 (26.6%) had MRSA. The majority (86.6%) of patients with *S. aureus* had moderate or mild SCORAD values. Only 12 (13.4%) patients had severe SCORAD values (Table 1).

**Table 1:**
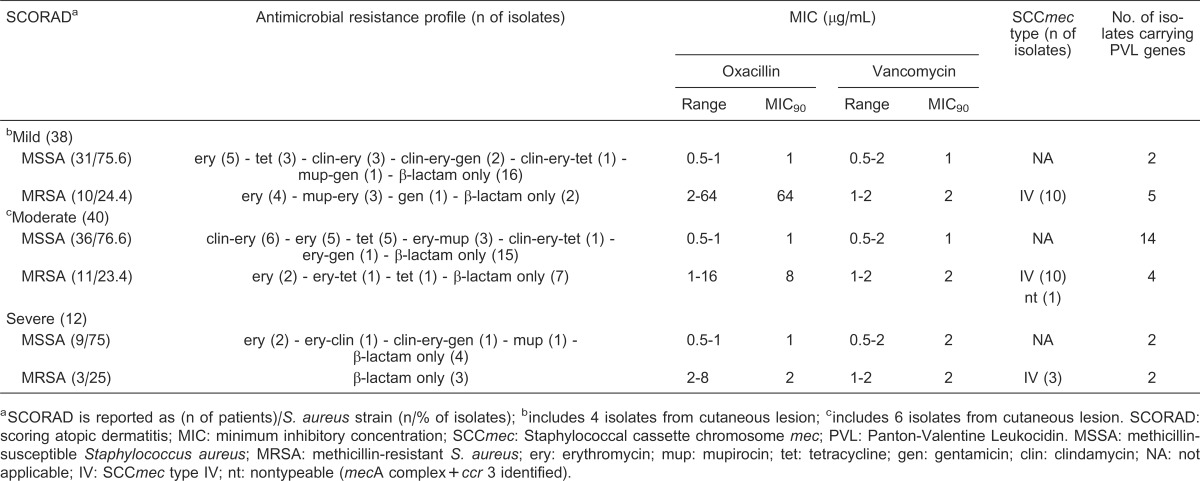
Characteristics of 100 Staphylococcus aureus isolates from nares and skin lesions from 90 pediatric patients with atopic dermatitis and correlation with SCORAD index

### Antimicrobial susceptibility and MIC

A total of 100 *S. aureus* isolates (90 from nares and 10 from skin lesions) were evaluated, and 24 were positive for MRSA. All isolates were susceptible to ciprofloxacin, chloramphenicol, linezolid, rifampicin, teicoplanin, tigecycline, and trimethoprim/sulfamethoxazole. Antimicrobial resistance was detected for erythromycin (40%), clindamycin (15%), tetracycline (12%), mupirocin (8%), and gentamicin (7%) (Table 1).

For oxacillin, 76% of the isolates in both nasal and skin sites presented MIC values between 0.5 and 1 µg/mL, but the values ranged from 2 to 64 µg/mL among the MRSA isolates (Table 1). For vancomycin, the values ranged from 0.5 to 2 µg/mL for all isolates. The eight mupirocin-resistant MRSA isolates presented high MIC values for mupirocin (≥1024 μg/mL).

### SCC*mec* typing and PVL genes

Among the 24 MRSA isolates, 23 (95.8%) carried the SCC*mec* IV, and 1 isolate was nontypeable and presented the complex A for the*mec* gene associated with the *ccr* 3 (Table 1).

Among 90 patients, 21 (23.3%) carried isolates with the PVL genes in the nares. Among the 8 patients with *S. aureus* isolates in their skin lesions, 6 (75%) possessed PVL genes. Among the children with moderate and severe SCORAD scores, 13 (32.5%) of 40 and 4 (33.3%) of 12 presented isolates positive for the PVL genes. Among patients with mild SCORAD, only 4 (10.5%) of the 38 presented this condition (P=0.0095). From the 100 *S. aureus* isolates evaluated, 18 (23.7%) of 76 methicillin-sensitive*S. aureus* (MSSA) and 11 (46%) of 24 MRSA were positive for the PVL genes.

### PFGE and MLST analysis

PFGE analysis showed that for 8 patients with at least two positive sites for*S. aureus*, 5 of them had isolates related to the USA1100/ST30 (3 patients) and USA800/ST5 (2 patients) lineages (Table 2). Two patients had isolates related to the USA400/ST1 lineage on skin lesions. The same lineages of*S. aureus* were found in the nares and skin lesions of four patients. All USA1100/ST30 and USA400/ST1 isolates were PVL positive.

**Table 2:**
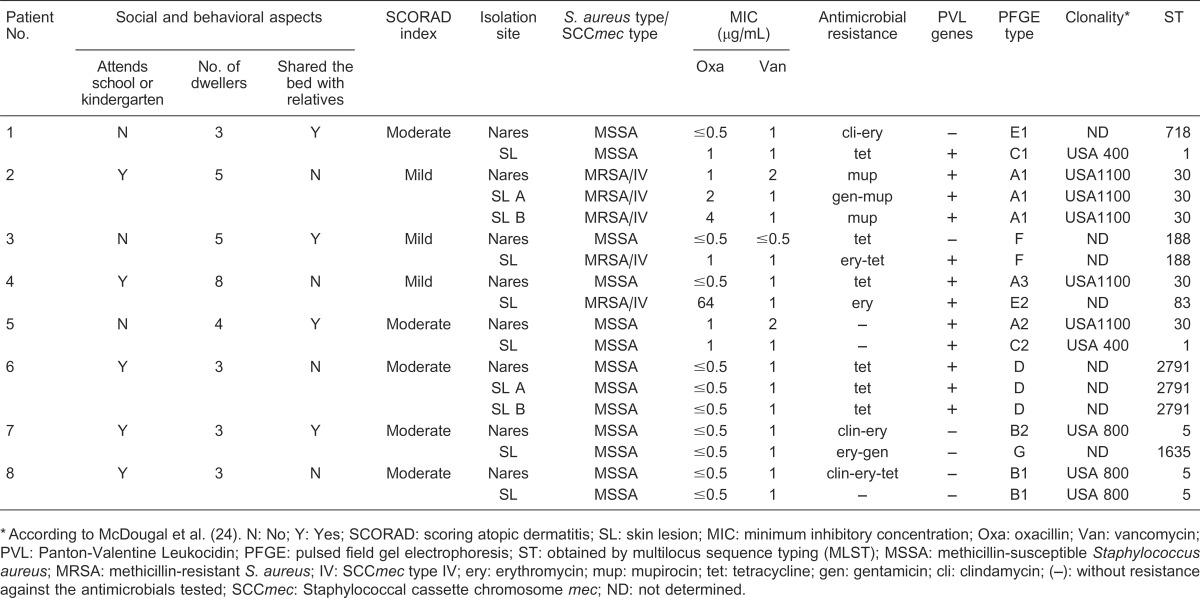
Characteristics of 18 Staphylococcus aureus isolates present in at least two clinical sites in 8 pediatric patients with atopic dermatitis.

Eight samples did not have profiles related to any previously described lineage. Among them, two isolates recovered from skin lesions belonged to ST83 and ST1635. One nare isolate was included in ST718. Two isolates (nasal and skin lesion) from the same patient belonged to ST188, but one of them was MRSA and the other was MSSA. A new sequence type, ST2791 (allelic profile 3-1-1-8-12-1-1) that differs from ST188 with an alteration in the *yqil* allele, was found associated with three isolates recovered from the same patient.

## Discussion

Various studies have characterized *S. aureus* isolates from AD patients (8,9,11-13,27,28). In this investigation, we found a very high prevalence of *S. aureus* among the AD patients. Out of 106 patients, 90 (85%) exhibited nasal colonization by this pathogen. Other studies have reported a prevalence of nasal carriers of up to 80% among AD children around the world (7-9). Graber et al. (9) conducted a study in children suffering from different chronic skin diseases and demonstrated that patients with AD were the most densely colonized with *S. aureus*. This might be related to the supposed role of *S. aureus* in the pathogenesis of this disease and/or may be associated with defective innate immunity in these patients (29).

Among the 90 patients colonized by *S. aureus* isolates, 86.6% presented mild or moderate AD (Table 1). Interestingly, Balma-Mena et al. (12) found that among 200 AD pediatric patients colonized by *S. aureus* who attended a dermatological outpatient clinic in Canada, 81% presented with the mild and moderate forms of AD, which is very similar to our findings. On the other hand, Pascolini et al. (13) verified that 77% of Italian children with high SCORAD values presented with *S. aureus*colonization, while only 15% of children had mild AD. Likewise, Rojo et al. (30) found a high prevalence rate of *S. aureus* among patients in Spain with moderate and severe AD. These conflicting results indicate that both the presence of the pathogen and its production of virulence factors may be relevant in AD aggravation.

In our study, MRSA isolates were detected in 26.6% of patients. However, studies in the literature have reported MRSA prevalence rates ranging from 0.5% to 16% in AD pediatric patients (7,9,12,13). The high level found in our study may be explained, in part, by the climatic characteristics of our country, the social aspects of the patients enrolled (largely low income), and the high prevalence of MRSA (7.5%) in the Brazilian healthy infant population (31). High methicillin resistance among *S. aureus* isolates can be worrying because these patients require aggressive antibiotic therapy and the β-lactam drugs are the first choice in AD staphylococcal infections. Furthermore, the high resistance level to mupirocin that was observed in all mupirocin-resistant MRSA isolates in this study may have prevented decolonization of these patients.

Among the MRSA isolates, 95.8% carried SCC*mec* IV. Likewise, Chung et al. (11) showed the predominance of SCC*mec* IV among isolates from pediatric AD patients in South Korea. However, Lo et al. (32) conducted a study with AD pediatric patients in Taiwan and identified SCC*mec* V as the prevalent cassette. As *S. aureus* belonging to ST59/SCC*mec* V is the most prevalent lineage in the Taiwan community (33), the lineage characteristics found in AD patients might be specific for each geographical region. This hypothesis could be supported by the PFGE and MLST analyses results of our isolates. Among 18*S. aureus* isolates evaluated by these methods, 10 (55.5%) belonged to the USA1100/ST30, USA400/ST1, and USA800/ST5 lineages that are normally associated with MRSA isolates in Brazil (16,22). Also, USA300/ST8 and the isolates from clonal complexes 5, 45, and 80 are frequently found in both AD patients and healthy community populations in the USA and Canada (9,28).

In the present study, five PFGE profiles belonging to ST83, ST188, ST718, ST1635, and ST2791 (a new sequence type related to ST188) were detected in isolates from five different patients. Among these lineages, only ST188 had been previously detected in Brazil among MSSA isolates recovered from hospitalized patients (22). Interestingly, ST188 was the most common lineage isolated from adults and adolescents with AD colonized by *S. aureus* in South Korea, accounting for 19.2% of the isolates (27). Furthermore, the authors also noted a wide diversity of lineages including sporadic and unusual clones among individuals with AD, which is similar to our findings.

Our molecular analysis of nares and skin lesion isolates recovered from the same patient showed that the isolates were identical in four of the eight (50%) cases. Other studies have also shown that the majority of *S. aureus*isolates recovered from the nares and skin lesions of the same pediatric AD patient exhibited the same genotypic profiles (9,13).

*S. aureus* is known to produce various potent toxins that can aggravate AD by triggering skin inflammation, and PVL is believed to play a key role in this recrudescence (6). In this study, PVL genes were detected in 29 isolates from 21 (23.3%) patients and were found in 75% of skin lesions. These findings differ from the majority of studies conducted in other countries that have found very low PVL rates ranging from 0% to 4.2% (11,13,28). However, Lo et al. (32) found that 71% of MRSA isolates recovered from skin lesions and nares of AD children in Taiwan were PVL positive. These authors detected isolates mainly belonging to ST59, a lineage strongly associated with PVL genes in that country, justifying its presence in AD patients (33). In the present study, all USA1100/ST30 isolates, a PVL-producing lineage prevalent in Rio de Janeiro (16), were positive for PVL genes. Furthermore, USA400/ST1 isolates were also positive for these genes, an unusual characteristic among isolates of this lineage in Brazil. Moreover, three isolates of a new lineage (ST2791) related to ST188 were also detected, and all of them were positive for PVL genes. This might be associated with the high occurrence of PVL-positive isolates recovered in this study.

Interestingly, we found that PVL genes were significantly more prevalent among children with moderate and severe SCORAD values (P=0.0095) compared to those with mild SCORAD values. Yeung et al. (28) evaluated 119 nasal and skin *S. aureus* isolates from adults and children with AD in Canada and did not find any obvious association between these genes and increased disease severity. Although further studies are necessary to elucidate the role of PVL in AD recrudescence, our data suggest that PVL might contribute to the greater severity of this skin disease among the children in this study.

Our results increase the understanding of MRSA epidemiology in AD patients and can help clinicians to design improved therapies. Children colonized by MRSA underwent decolonization with topical mupirocin, except for those carrying mupirocin-resistant isolates, which were treated with trimethoprim/sulfamethoxazole (data not shown). Some authors have shown that community-acquired MRSA isolates are susceptible to trimethoprim/sulfamethoxazole (34) and have suggested the use of this drug as an option in MRSA decolonization schemes (35,36). These data are in agreement with our study showing that all MRSA isolates were trimethoprim/sulfamethoxazole-susceptible. Thus, patients with cutaneous MSSA and MRSA infections were successfully treated with cefalexin and trimethoprim/sulfamethoxazole, respectively.

This study showed a high prevalence of *S. aureus* and MRSA recovered from pediatric patients with AD in Brazil, including emergent lineages. We also found a high frequency of PVL genes among severe and moderate SCORAD patients. These factors may affect lesion severity and thus may contribute to improvements in the management policies of pediatric AD patients.
